# Genetic Divergence and Signatures of Natural Selection in Marginal Populations of a Keystone, Long-Lived Conifer, Eastern White Pine (*Pinus strobus*) from Northern Ontario

**DOI:** 10.1371/journal.pone.0097291

**Published:** 2014-05-23

**Authors:** Vikram E. Chhatre, Om P. Rajora

**Affiliations:** 1 Department of Biology, Dalhousie University, Halifax, Nova Scotia, Canada; 2 Faculty of Forestry and Environmental Management, University of New Brunswick, Fredericton, New Brunswick, Canada; North Carolina State University, United States of America

## Abstract

Marginal populations are expected to provide the frontiers for adaptation, evolution and range shifts of plant species under the anticipated climate change conditions. Marginal populations are predicted to show genetic divergence from central populations due to their isolation, and divergent natural selection and genetic drift operating therein. Marginal populations are also expected to have lower genetic diversity and effective population size (*N*_e_) and higher genetic differentiation than central populations. We tested these hypotheses using eastern white pine (*Pinus strobus*) as a model for keystone, long-lived widely-distributed plants. All 614 eastern white pine trees, in a complete census of two populations each of marginal old-growth, central old-growth, and central second-growth, were genotyped at 11 microsatellite loci. The central populations had significantly higher allelic and genotypic diversity, latent genetic potential (LGP) and *N*_e_ than the marginal populations. However, heterozygosity and fixation index were similar between them. The marginal populations were genetically diverged from the central populations. Model testing suggested predominant north to south gene flow in the study area with curtailed gene flow to northern marginal populations. Signatures of natural selection were detected at three loci in the marginal populations; two showing divergent selection with directional change in allele frequencies, and one balancing selection. Contrary to the general belief, no significant differences were observed in genetic diversity, differentiation, LGP, and *N*_e_ between old-growth and second-growth populations. Our study provides information on the dynamics of migration, genetic drift and selection in central versus marginal populations of a keystone long-lived plant species and has broad evolutionary, conservation and adaptation significance.

## Introduction

Marginal populations are expected to provide the frontiers for adaptation, evolution and range shifts of plant species under the anticipated climate change conditions. Marginal populations are generally adapted to their sub-optimal habitats, and are predicted to be genetically diverged from central populations due to their isolation, and divergent natural selection and genetic drift operating therein [1], [2]. The central-marginal hypothesis proposes that populations at the species’ range peripheries have lower genetic diversity and higher genetic differentiation than populations at the species’ center of abundance. This may in-part result from differential selection regimes, isolation, higher stochastic genetic drift, restricted gene flow, smaller census and effective population size (*N*_e_) and sub-optimal habitats in marginal populations [1], [2], and may consequently lead to genetic divergence from central populations. At range peripheries, organisms may experience a host of harsh environmental, climatic, edaphic, and nutrient conditions, and impediments to gene flow. In such environments, selection regimes different from those at the abundant center may operate. This may drive allele frequencies for selected genes, ultimately resulting in local adaptation. Thus, marginal populations are important for future evolution and adaptation of species and may serve as grounds for speciation [1], [3]. On the other hand, intense competition for resources and abiotic stresses at the leading edge may cause marginal populations to have negative growth rates [4] and to become demographic sinks with reduced fecundity [3], [5], as has been shown in lodgepole pine (*Pinus contorta*) [6]. Evolutionary success or failure of populations at range margins is also dependent upon the balance between gene flow from the abundant center and local adaptation [4], [7]. For example, asymmetrical abundant gene flow from central to marginal populations can result in maladaptation of populations at the range margins due to the infusion of maladaptive alleles from the center [7]. Naturally, the conservation significance and local adaptation of marginal populations have been debatable and depend upon their evolutionary potential for adaptation [1], [3], [8], particularly in the face of rapidly changing climate, emergence of new diseases and loss of habitat [9]. Therefore, it is crucial to understand patterns of genetic diversity, population structure and evolutionary processes such as natural selection, genetic drift and gene flow in central versus marginal populations, and genetic mechanisms underlying local adaptation in marginal populations, especially in long-lived, widely-distributed keystone plant species. This information is critical for forest trees as they are normally the keystone species in their ecosystems. Existence and survival of many flora and fauna in an ecosystem depend upon the existence of such keystone species.

While a number of studies have tested the central-marginal hypothesis in a diverse group of plants and animals, including forest trees [1], [2], the issue remains unresolved for the lack of overwhelming evidence in favor of it. Forest trees, especially conifers, are particularly suitable for testing various central-marginal hypotheses, because of their longevity and wide geographical distribution accounting for environmental, ecological and selection heterogeneity over time and space. A number of studies in conifers have supported the central-marginal hypothesis, e.g., [10]–[12]; while many others did not, e.g., [13]–[15]. In recent years, landscape genetics of range margins has seen renewed interest, especially to understand local adaptation of marginal populations [16], [17]. As a result, reports on detection of divergent selection in marginal populations of forest trees [18] and other plants [19] are beginning to emerge. However, the information is very scarce. We are not aware of any report on natural selection in *in-situ* natural marginal versus central populations of forest trees. Holliday *et al*. study [18] was conducted on samples from a common garden provenance test.

In long-lived plants, such as conifers, there is a general belief that old-growth populations harbor higher genetic diversity than second-growth populations. However, empirical data to support or contradict this view is scarce [20], [21]. Nevertheless, it is important to take the population age into account when comparing central and marginal population genetic characteristics.

Eastern white pine (*Pinus strobus* L.) provides an ideal organism to test the central- marginal and old-growth second-growth hypotheses. It is an ecologically important keystone species of white pine ecosystems, economically important for timber production, and long-lived (>400 years), with wide geographical distribution in North America [22], [23]. Its natural range extends in southern Canada from Newfoundland to extreme southeastern Manitoba to southeast to northern Georgia and northwestern South Carolina [22]. In the pre-colonial era, eastern white pine covered much of the eastern North America [22]. However, it has undergone heavy exploitation during and after the colonial era [23], and post-glacial range expansion and retraction [24]. Much of the original old growth eastern white pine has vanished [23]. Ontario and Quebec still have some eastern white pine old-growth stands, where it exists mostly as second-growth forest. The range of eastern white pine is expected to shift northwards under anticipated climate change conditions, such as in northern Ontario.

Eastern white pine (EWP) is a predominantly outcrossing species [14], [25] and has high inbreeding depression. It has moderate to high levels of genetic diversity [13], [26]–[30]. Rajora *et al*. [13], [14] reported similar levels of allozyme-based genetic diversity, population structure and outcrossing levels in EWP from central Ontario and about 2,000 km apart disjunct marginal Newfoundland populations, although inbreeding levels estimated from the empty seed data were higher in the Newfoundland populations. However, there is a lack of information on genetic diversity and differentiation of central and marginal populations from the same regional part and entire natural range, as well as on any selection regimes underlying local adaptation that may be operational in marginal populations of this species. Also, information on genetic diversity in old-growth versus second-growth populations is scarce [28].

Here, we address the hypothesis that geographically marginal populations of eastern white pine have lower genetic diversity and higher genetic differentiation than and are genetically distinct from geographically central populations. We also asked the question whether old-growth populations have higher genetic diversity than second-growth populations. We undertook comprehensive genetic diversity and population structure analyses in a complete census of four old-growth and two second-growth populations of eastern white pine from its central and marginal distribution in northern Ontario, Canada. We also tested various models for gene flow between populations, estimated *N*_e_, and tested loci for signatures of natural selection.

## Materials and Methods

### Ethics Statement – Field Sampling

The studied eastern white pine populations are located on public lands in Ontario, Canada, which are not designated as protected areas. The field sampling was done by the employees of the Ontario Ministry of Natural Resources, which manages public forests there. Therefore, no specific permission was required for field sampling from the studied locations. Also, our study did not involve any endangered species.

### Study Populations, Experimental Design and Sampling

**Figure 1:**
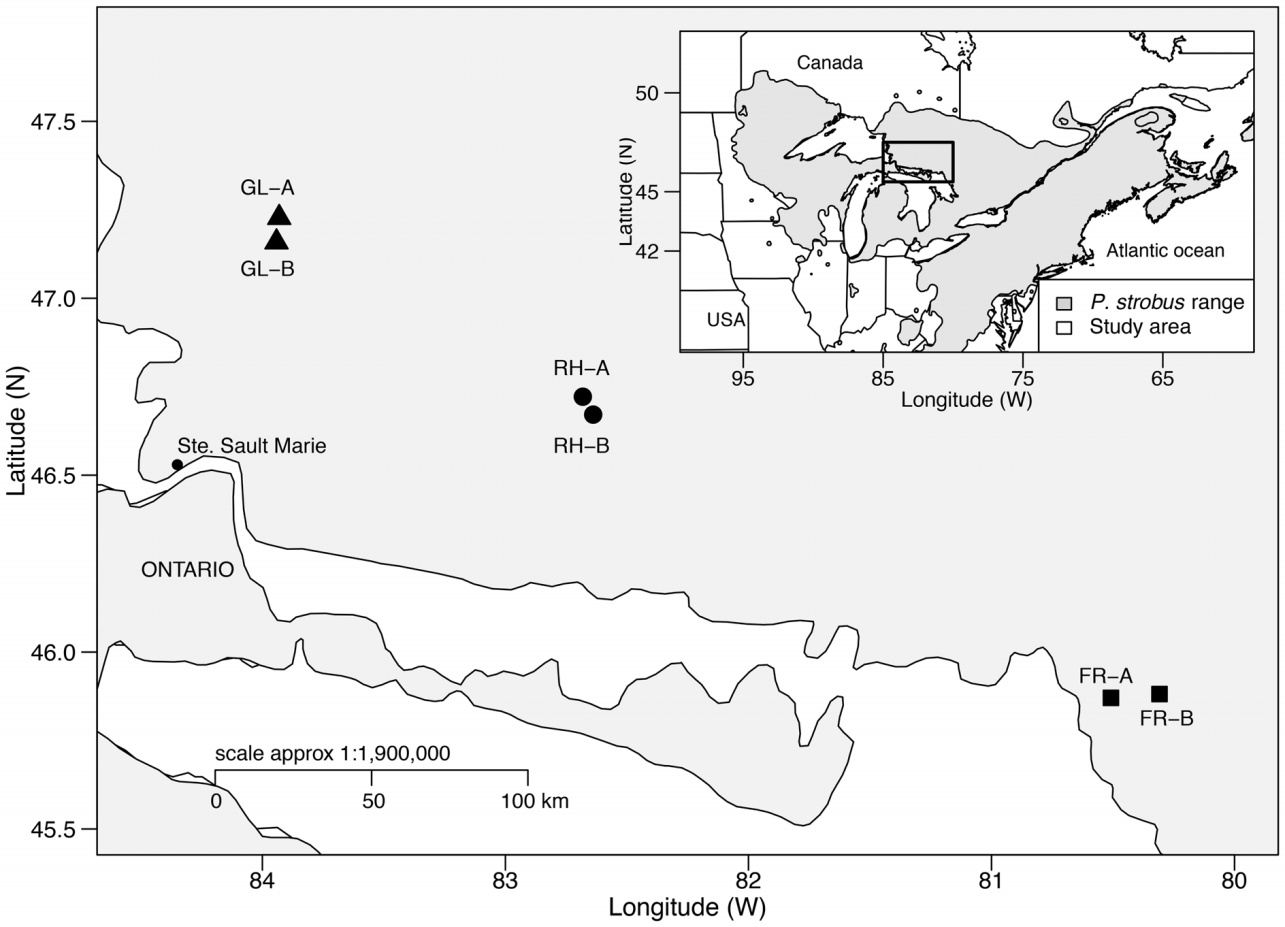
Eastern white pine distribution range and location of six studied populations.

We studied six EWP natural populations from its natural distribution range in northern Ontario, Canada (Fig. 1; Table 1). While the French River (FR) and Rawhide Lake (RH) stands are part of the continuously distributed EWP central populations, Galloway Lake (GL) populations are located at the species’ northern boundary and are somewhat geographically isolated (Fig. 1). The average age of the populations at sampling was: GL –250 years; RH –235 years; and FR –100 years.

**Table 1:** Eastern white pine populations studied, their sizes and geographical coordinates.

Population	Pop ID	Region	Pop Type	Size	Latitude (N)	Longitude (W)
Rawhide Lake A	RH-A	Central	Old-growth	99	46 40′ 16″	82 38′ 18″
Rawhide Lake B	RH-B	Central	Old-growth	97	46 40′ 18″	82 37′ 16″
French River A	FR-A	Central	Second-growth	100	45 52′ 14″	80 28′ 42″
French River B	FR-B	Central	Second-growth	94	45 52′ 53″	80 21′ 31″
Galloway Lake A	GL-A	Marginal	Old-growth	122	47 13′ 40″	83 55′ 50″
Galloway Lake B	GL-B	Marginal	Old-growth	102	47 13′ 07″	83 56′ 30″

Pop = Population.

We chose to conduct an in-depth genetic analysis for a fewer populations from the same regional part of the natural distribution range of EWP in northern Ontario; first because the northern populations in this part of the EWP range are expected to shift range northwards and second the selection pressures in different parts of the marginal range of this species are likely to be different. Two populations in one area separated by 2 to 10 km, were studied as replicates of a population type (marginal, central, old-growth, second-growth), in order to minimize confounding effects of major eco-site and climate conditions by sampling far-distant populations from a different part of the species range. In order to minimize the effect of population age on central-marginal comparisons, we sampled two old-growth (OG) marginal, two old-growth central and two second-growth (SG) central populations. We could not locate second-growth marginal populations in our study area. We sampled all trees in a complete census of the marginal populations from the GL area [27], [31]. In the continuous RH and FR populations, plots of the same number of trees (∼100) were established to keep the sampling uniform across the populations. All trees in the established plots were censused, so as to minimize missing rare alleles, which may be a subject or product of natural selection and/or genetic drift and contribute to the latent genetic potential [32].

The two populations from the Rawhide Lake area are located 30 km north of Elliot Lake, Ontario, in the Rawhide Lake Conservation Reserve established in 2000 by Ontario Ministry of Natural Resources (OMNR). This area lies to the north of Mississagi Provincial Park. Classified as ecological site 5E, it is characterized by rocky landscape rich in glacial history and Moraine deposits. Old and ecologically mature pure eastern white pine stands are located in the western part of the reserve and are interspersed with sugar maple (*Acer saccharum*) and jack pine (*Pinus banksiana*) dominated stands (Information source: Rawhide Lake Conservation Reserve Fact Sheet, OMNR, 2000).

The two populations from the French River area are located near Grundy Lake Provincial Park in Ontario, Canada, occupying an area that was glaciated during Pleistocene and was subsequently dominated by EWP. Historical documents indicate three different periods of logging in this general area, the first of which was around 1870s by the Victoria Timber Company, which exported timber to the United States. The subsequent harvesting of mature eastern white pines occurred in two episodes starting in 1900 and 1930 respectively. The French River area population A is of post-fire origin, whereas French River B of post-harvest origin (Dianne Othmer, OMNR, pers. comm.).

The Galloway Lake old pine area, located roughly 100 km north of Sault Ste. Marie, Ontario, hosts the two old-growth EWP populations used in this study. This area, classified as ecosite 4E [33], has complex topography consisting of rivers and lakes interspersed with rocky areas, with soil texture varying between fine sands and silt loams, and variable depth [23]. The mature EWP stands in this area are believed to have post-fire origin, mixed with hardwood species. The study populations A and B were small and had eastern white pine individuals of about 250 years, mixed with a variety of hardwood and conifer understory species [31].

### Microsatellite Genotyping

We genotyped 390 trees from the four central populations at 11 nuclear microsatellite loci (Table S1) developed by Echt *et al*. [34]. The genotype data for 224 trees from the marginal GL populations was obtained from Rajora *et al.* [27]. To attain complete allele-size correspondence between RH, FR and GL datasets, we genotyped 10 individuals from each population in the same single run for each of the 11 microsatellites and confirmed that individual allele sizes were consistently identified correctly in all six populations. All microsatellite loci were in linkage equilibrium. The details of microsatellite genotyping methods, including PCR conditions, are provided in the Microsatellite Genotyping Text S1.

### Data Analysis

Data analyses were performed in three sets: all six populations; central versus marginal populations; old-growth versus second-growth populations.

#### Genetic diversity

Standard measures of genetic diversity were calculated using GENEPOP [35]. In addition, number of private alleles per population, latent genetic potential (LGP: difference between effective and mean number of alleles over all loci) [36], and observed and expected genotypic additivity (richness) [27] were also calculated. Inbreeding coefficient was determined using Weir and Cockerham’s estimate of Wright’s *F*_IS_ [37]. Significance of differences in genetic diversity estimates between groups of populations was tested using ANOVA and Duncan’s Multiple Range Test (DMRT) in the R environment [38].

### Population Structure

The genetic structure of the studied populations was examined using several complementary methods: single-locus and multilocus *F*_ST_ using Weir and Cockerham’s method [37] in GENEPOP [35]; hierarchical AMOVA [39] in GENALEX [40]; and Bayesian modeling using STRUCTURE [41]. Nei’s [42] genetic distances between individual and groups of populations were calculated. To determine if *F*_ST_ between marginal populations was significantly higher than that between central populations, we applied one-sided and two-sided significance tests with 1000 permutations using FSTAT [43]. Significance of hierarchical AMOVA was tested with 999 permutations in GENALEX [40].

Allele frequency heterogeneity among pairs of populations was tested using Fisher’s exact test in GENEPOP [35]. Genetic relationships among populations were resolved using the Neighbor-Joining (NJ) method [44] in POPTREE [45]. We employed 5×10^5^ bootstrap iterations for testing statistical support for clustering. Principal coordinate analysis (PCA) based on Nei’s [42] genetic distances was performed and the ordination of the populations on the first two principal components was plotted using GENALEX [40].

Bayesian analysis of the population structure was performed using the admixture model in STRUCTURE [41]. We assumed that all markers were unlinked (as they actually were), all trees were admixed and that allele frequencies were correlated between loci. The number of genetic clusters (*K*) tested varied from 1 through 6 when all six populations were analyzed, and from 1 through 4 when only central OG and SG populations were analyzed. We discarded the first 10^5^ steps of each run as burn-in, to allow the parameters to converge, prior to collecting the data for the next 10^5^ steps. Different combinations of burn-ins and MCMC chains (between 0.25×10^5^ and 1×10^5^) were performed initially to find out the point of convergence for our data set and 1×10^5^ steps of burn-in and MCMC chains each, per run were found to be sufficient. Twenty-five such runs for each putative value of *K* were performed to test for variance between the runs. To estimate the most optimal value of *K* for each level of population comparisons, we further processed the program output through STRUCTURE HARVESTER [46], which employs the Δ*K* ad-hoc statistic of [47]. Once an optimal number of clusters was inferred, we performed 10^3^ permutations for each of the 25 replicates of the chosen *K* using the Greedy algorithm in CLUMPP [48], in order to match the replicates as closely to each other as possible. Finally, barplots of cluster membership assignments were prepared with DISTRUCT [49].

Isolation by distance was tested by regressing the logarithm of pair-wise geographic distance with pair-wise *F*_ST_/1– *F*_ST_ using Mantel test over 1,000 permutations. Additionally, to account for the bias introduced by hierarchical population structure, stratified and partial Mantel tests were performed using genetic clusters and geography as covariates following [50] using VEGAN R package [51].

#### Signatures of natural selection

We employed *F*_ST_ outlier approach in LOSITAN [52], [53] to identify loci deviating from neutrality and potentially under selection. Mean neutral *F*_ST_ was estimated and applied for 5×10^5^ simulations to construct upper and lower 95% confidence limits. Markers with unusually high or low *F*_ST_ violating these thresholds were identified as candidates for selection. This analysis was done for three sets: all six populations, four central populations and three location-wise pooled populations. A built-in false positive correction was applied using a false discovery rate of 0.05.

Assuming the finite island model for detecting loci under selection can result in a large proportion of false positives due to confounding effects of shared history of populations and population structure or if the population samples are derived from hierarchically subdivided populations [54]. In such cases, a hierarchical island model [55] is better suited. In the hierarchical island model, Wright’s *F*-statistics is described by *F*_ST_ as variance among populations in-total and *F*_CT_ as variance among regions (central and marginal in this study) [54]. We used this model for detecting outlier loci potentially under selection using hierarchical *F*-statistics analysis following [54] by performing 1×10^5^ coalescent simulations in ARLEQUIN [56]. This model requires that the number of groups (regions) be equal to or larger than the actual number sampled and that the number of demes (populations within a region) simulated be more than the actual number sampled [54]. To satisfy these conditions, we chose 20 groups and 100 demes per group for the simulations. The resulting 1×10^5^ hierarchical fixation indices were used as null distributions to construct a 95% confidence interval and to identify loci outside the two-tailed significance threshold of *P* = 0.05, as outliers.

For comparison, we also employed a Bayesian method to detect outlier loci based on differences in allele frequencies between four central and two marginal populations using the software BAYESCAN [57]. Analysis consisted of 50 pre-calculation pilot runs, each spanning 20,000 steps. The calculation stage included an initial burn-in period of 2×10^5^ steps followed by data collection for the next 10^5^ steps. Posterior probability q values (FDR corrected *P* values) were used to assign outlier status to markers. We performed a Genbank protein homology search (BlastX) to functionally annotate the sequences from which the markers found under natural selection were developed. The direction of selection on loci showing divergent selection was assessed by allele frequency changes in marginal populations.

#### Gene flow and effective population size

Bidirectional gene flow between pairs of populations and mutation-scaled *N*_e_ were estimated using MIGRATE software [58]. This method assumes roughly equal distance between populations. Due to proximity of individual populations from the same study area, we selected one of the two study sites at each location as a representative sample for estimating gene flow. Bayesian model testing approach was applied to find the model that best explains the observed data at the three sampling locations. Three models were tested: north to south, south to north, both with unrestricted gene exchange between the two central populations, and a full symmetrical migration matrix encompassing all possible routes between all pairs of populations. Initial 20,000 Markov chain sweeps per locus were discarded as burn-in and data was then collected for another 20,000 sweeps at an increment of 100 sweeps. The analysis was repeated five times and estimates were combined over replicates. Relative geographical distance between pairs of populations was taken into consideration when estimating the rate of migration and θ. The number of migrants per generation was estimated by multiplying parameter *M* [58] by θ. Mutation-scaled effective population sizes (*N*_e_) for the six individual populations were estimated from a separate MIGRATE analysis under the following conditions. Two loci, RPS-20 and RPS-39 were removed from the analysis due to their *F*_ST_ outlier status and 50 samples were randomly chosen per population. Ten short Markov chains and one long Markov chain were sampled per locus with the first 50,000 trees discarded as burn-in and the next 50,000 trees recorded over two replicates. Static heating was used and chains were allowed to swap. In addition, two replicate populations at each of the three sampling locations were combined into a pooled data set which was also analyzed with MIGRATE (50,000 trees discarded; 50,000 trees recorded). The *N*_e_ was then estimated for individual and pooled populations from the θ parameter as follows: since θ = 4N_eµ_, therefore, *N*_e_ = ^θ/^4×_µ_. The mutation rate (_µ_) was assumed to be constant at 10^–3^ for all microsatellite loci [11], [12], [59]. To resolve the effect of environmental conditions on the dynamics of gene flow, information on historical wind patterns in the study area was obtained from the Atlas of Canada [60].

## Results

### Genetic Diversity, Fixation Index, Latent Genetic Potential, and *N*_e_

The results on genetic diversity measures, *F*_IS_, LGP and *N*_e_ for individual populations and means for central and marginal, OG and SG populations are reported in Table 2. The central SG population FR-A had the highest and the marginal OG population GL-B the lowest allelic and genotypic diversity and LGP (Table 2). In contrast, the marginal OG GL-B population had the highest and the central FR-B SG population the lowest observed (*H*_o_) and expected (*H*_e_) heterozygosity (Table 2). The central populations had 30 and marginal populations 6 private alleles (*A*_P_). The central populations had significantly (*P*<0.05) higher allelic diversity (*A*_T_, *A* and *A*_R_), *A*_P_, expected genotypic additivity, and LGP than the marginal populations. However, the marginal populations had higher heterozygosity than the central populations but the differences were not significant (*P*>0.05).

**Table 2:** Genetic diversity parameters, fixation index and effective population size of eastern white pine populations.

Region	Type	Pop	*A*_T_	*A*	*A*_e_	*A*_P_	*A*_R_	*H*_o_	*H*_e_	*F_IS_*	LGP	*GA*_o_	*GA*_e_	*N*_e_
**Individual populations**														
Central	OG	RH-A	130	11.82	4.23	6	11.55	0.535	0.603	0.113	83.49	246	1226	312.3
		RH-B	129	11.73	4.22	10	11.57	0.496	0.608	0.183	82.56	264	1046	328.5
	SG	FR-A	133	12.10	4.50	9	11.83	0.531	0.612	0.133	83.53	284	1242	400.5
		FR-B	113	10.27	3.59	5	10.17	0.457	0.557	0.181	73.54	210	956	353.1
Marginal	OG	GL-A	104	9.45	3.86	3	9.24	0.538	0.626	0.142	61.57	225	733	235.1
		GL-B	102	9.27	4.40	3	9.12	0.590	0.644	0.084	53.55	217	701	211.9
**Overall mean**			118.5	10.77	4.13	4.3	10.58	0.525	0.608	0.139	73.04	241	984	306.7
**Population means**														
***Central vs Marginal***														
Central	OG+SG		126^A^	11.48^A^	4.13^A^	7.5^A^	11.28^A^	0.503^A^	0.594^A^	0.153^A^	80.78^A^	251^A^	1117^A^	348.6^A^
Marginal	OG		103^B^	9.37^B^	4.13^A^	3^A^	9.18^B^	0.564^A^	0.635^A^	0.113^A^	57.56^B^	221^A^	717^B^	223.5^B^
***Central OG vs Central SG***														
Central	OG		130^A^	11.78^A^	4.22^A^	8^A^	11.56^A^	0.513^A^	0.605^A^	0.148^A^	83.03^A^	255^A^	1136^A^	320.4^A^
Central	SG		123^A^	11.18^A^	4.04^A^	7^A^	11.00^A^	0.492^A^	0.583^A^	0.157^A^	78.53^A^	247^A^	1099^A^	376.8^A^
**Central OG ** ***vs*** ** Marginal OG**														
Central	OG		130^A^	11.78^A^	4.22^A^	8^A^	11.56^A^	0.513^A^	0.605^A^	0.148^A^	83.03^A^	255^A^	1136^A^	320.4^A^
Marginal	OG		103^B^	9.37^B^	4.13^A^	3^A^	9.18^B^	0.564^A^	0.635^A^	0.113^A^	57.56^B^	221^A^	717^B^	223.5^B^

Pop = Population; OG = Old-growth; SG = Second-growth; *A*_T_ = Total number of alleles; *A* = Mean number of alleles per locus; *A*_e_ Mean effective number of alleles per locus; *A*_p_ = Number of private alleles; *A*_R_ = Allelic richness; *H*_o_ = Mean observed heterozygosity; *H*_e_ = Mean expected heterozygosity; *F*is = Mean fixation index; LGP = Latent genetic potential; *GA*o = Observed genotypic additivity; *GA*e = , Expected genotypic additivity; *N*_e_ = Effective population size. Means followed by different letters are significantly different at *P*<0.01, based on ANOVA and Duncan’s multiple range test (DMRT).

The mean *F*_IS_ ranged from 0.084 in GL-B to 0.183 in RH-B, with a mean of 0.139 over all populations. On average, *F*_IS_ was lower in the marginal than central populations, but the differences were not significant (Table 2). Similar patterns of genetic diversity and LGP measures for central versus marginal populations were observed when we compared only two central OG populations with two marginal OG populations (Table 2). The central old-growth and central second-growth populations showed statistically similar allelic and genotypic diversity, LGP and *F*_IS_ (Table 2).

*N*_e_ ranged from 212 in the marginal OG GL-B population to 401 in the central SG FR-A population, with an overall mean of 307 (Table 2; Fig. S8). The central populations had significantly higher *N*_e_ than the marginal populations (*P*<0.01), whereas central OG and central SG populations showed statistically similar *N*_e_ (Table 2; Fig. S8). The confidence intervals for *N*_e_ estimates did not overlap between the central and marginal populations (Fig. S8). Pooled population analysis also showed higher *N*_e_ in the central populations (RH: 401; FR: 398) than in the marginal population (GL: 312). All *N*_e_ estimates were within the bounds of 95% confidence intervals, which again did not overlap between the central and marginal populations (Fig. S8).

### Population Genetic Structure and Differentiation

Well-defined population genetic structure was observed among the six populations, where marginal populations showed significant genetic divergence from the central populations. The multi-locus *F*_ST_ ranged from 0.03 between RH-A and FR-A to 0.134 between FR-B and GL-B, with an overall mean of 0.083 among all six populations (Table S2; Table 3). The *F*_ST_ estimates between the central and marginal populations were high and highly significant (Table 3), whereas between central OG and SG populations were low but still significant. Although *F*_ST_ among central populations (0.008) was lower than that between two marginal populations (0.021), the differences were not statistically significant (one-tailed *P* = 0.42, two-tailed *P* = 0.99). The genetic structure and differentiation patterns from hierarchical AMOVA mirrored those observed from *F*_ST_, confirming highly significant genetic differentiation between marginal and central populations (Table 3). Fisher’s exact tests revealed highly significant allele frequency heterogeneity between central and marginal populations (data not shown).

**Table 3:** Genetic differentiation of eastern white pine populations from *F*_ST_ and hierarchical AMOVA analyses.

Region	*F*_ST_	*P*	Φ_PT_/Φ_RT_	*P*
Central	0.008	0.001	0.014	0.001
Central OG	0.006	0.002	0.010	0.001
Central SG	0.010	0.001	0.017	0.001
Marginal	0.021	0.001	0.038	0.001
Central – Marginal	0.104	0.001	0.169	0.001
Central OG – SG	0.005	0.001	0.008	0.001
All populations	0.083	0.001	0.137	0.001

OG = Old-growth; SG = Second-growth.

Genetic distances between central and marginal populations were orders of magnitude higher (0.22 to 0.26) than among central populations (0.006 to 0.016) (Table S2). The NJ tree and PCA from Nei’s [42] genetic distances clustered six populations into two distinct groups, separating the marginal from the central populations (Figs. S1, S2). No particular grouping of OG versus SG populations was observed, when the analyses were performed for only central populations (data not shown).

**Figure 2:**
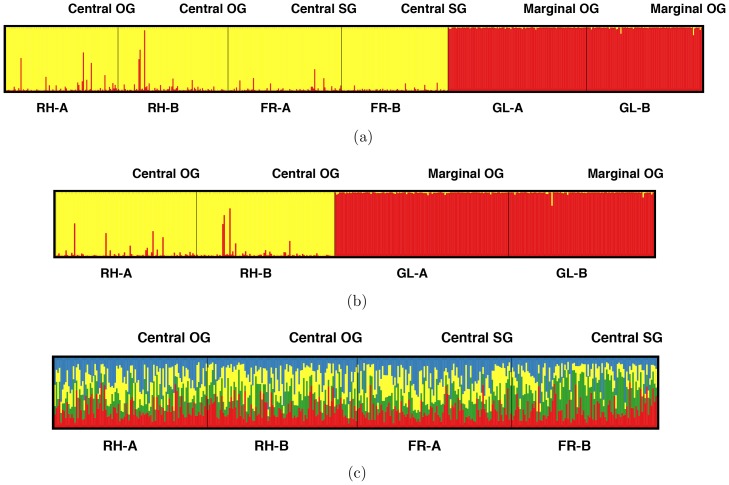
Population structure in central and marginal populations of eastern white pine. Bar plot of estimated membership coefficient (Q) of eastern white pine individuals from (**A**) all six central and marginal populations showing two groups corresponding to central and marginal populations at *K* = 2, (**B**) two marginal old-growth and two central old-growth populations showing two groups corresponding to central and marginal populations at *K* = 2, and (**C**) two central old-growth and two central second-growth populations showing no separate groups at *K* = 4.

The Bayesian STRUCTURE analysis identified two distinct clusters (*K* = 2) (Fig. S3) among six populations; clearly separating marginal populations from central populations (Fig. 2A). Individuals had more than 95% membership in their assigned cluster (Fig. 2A). Results from an independent analysis using only OG central and OG marginal populations also yielded highly similar results, with *K = *2, differentiating the marginal from central populations (Fig. 2B). When population structure was analyzed for central OG and SG populations using identical conditions, the Δ*K* suggested the optimal number of clusters to be four. The membership assignments of individuals across these four populations showed admixture among the four clusters (Fig. 2C). Thus, we can infer that the central OG and SG populations exhibit weak or no population structure.

Various Mantel tests for isolation by distance were not significant (regular Mantel test: *P* = 0.06; stratified Mantel test: *P* = 0.09; Mantel test for hierarchical population structure using genetic clusters: *P* = 0.09; and partial Mantel test with geography as covariate: *P* = 0.07).

### Signatures of Natural Selection

**Figure 3:**
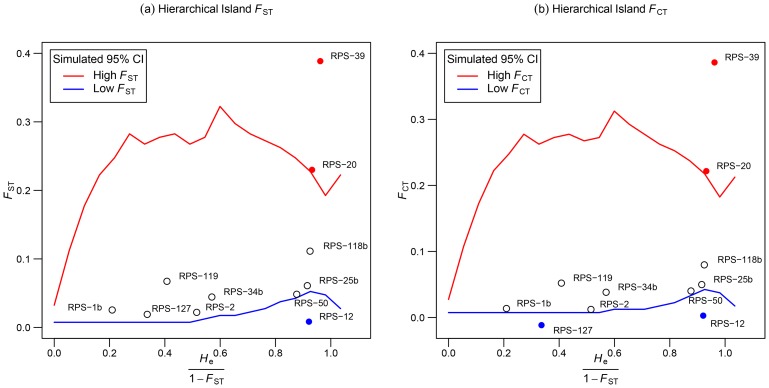
Loci showing signatures of natural selection under hierarchical island model. Outlier microsatellite loci under natural selection in eastern white pine populations with respect to hierarchical population structure as defined using a hierarchical island model.

**Figure 4:**
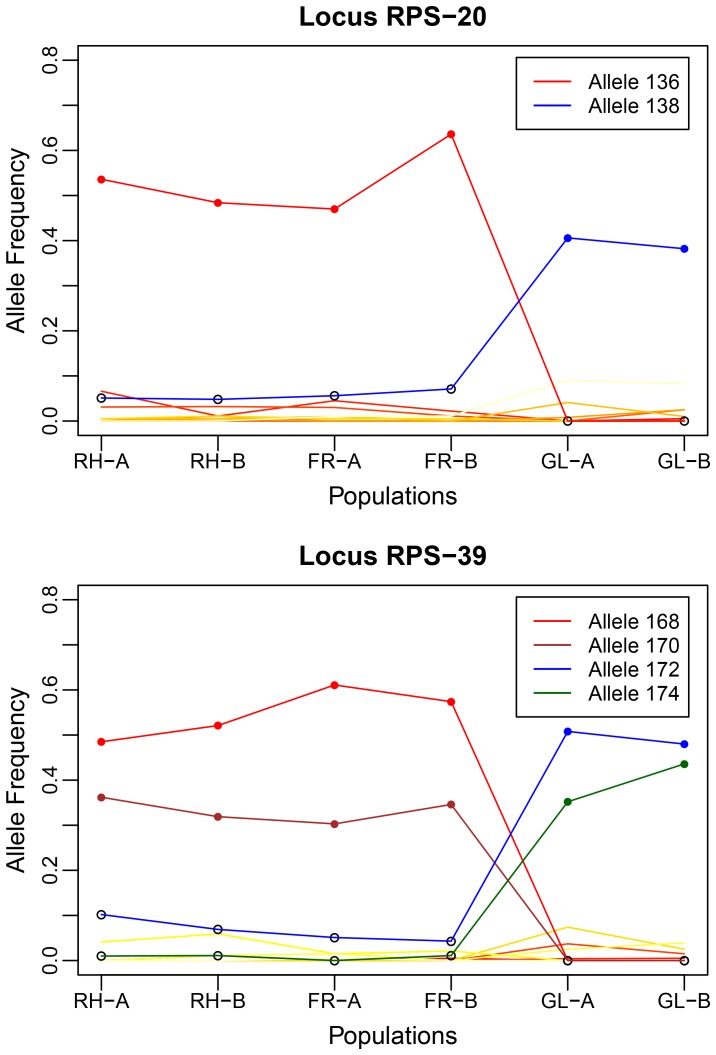
Allele frequency distribution at loci under divergent selection in marginal populations of eastern white pine.

The *F*_ST_ outlier test for six populations identified 5 loci violating the 95% confidence interval expectations under neutrality (Fig. S4). Two of these loci (RPS-20 and RPS-39) were candidates for divergent and three (RPS-12, RPS-25b and RPS-50) for balancing selection (Fig. S4a). When only central OG and marginal OG populations were tested, the results were mostly similar except that RPS-25b and RPS-50 were not candidates for balancing selection (Fig. S5). The hierarchical *F*_ST_ analysis with all six populations also confirmed these results where RPS-20 and RPS-39 loci were detected as candidates for divergent selection (Fig. 3); however, only RPS-12 was detected as candidate for balancing selection. One more locus RPS-127 was identified as an outlier potentially under balancing selection when *F*_CT_ was used to detect outliers in place of *F*_ST_ from the hierarchical analysis (Fig. 3). In contrast, no loci showed signatures of selection when only central group of OG and SG populations were compared (Fig. S4b). At RPS-20 and RPS-39, several high frequency alleles were either exclusive to central or marginal populations or showed significant directional change in the marginal populations (Fig. 4). When two populations were pooled for each of the three study locations, the number of outlier loci decreased to two (divergent candidate: RPS-39; balancing candidate: RPS-12) (Fig. S6). Bayesian analysis using BAYESCAN showed very similar results (Fig. S7). Four loci were identified as outliers, with RPS-39 reconfirmed as putatively being under diversifying selection and RPS-12 and RPS-50 under balancing selection. In addition, locus RPS-2 was also identified as balancing selection candidate. Overall, RPS-39 and RPS-20 were consistently identified as candidate microsatellite loci under divergent selection and RPS-12 as a candidate for balancing selection by almost all analyses. Thus, we consider only these three microsatellite loci as putative loci under selection in the marginal populations. RPS-20 showed significant homology to a hypothetical protein of unknown function, and RPS-12 to KAOT1–09806 and EAI-06382 proteins (Table S3).

### Gene Flow

**Figure 5:**
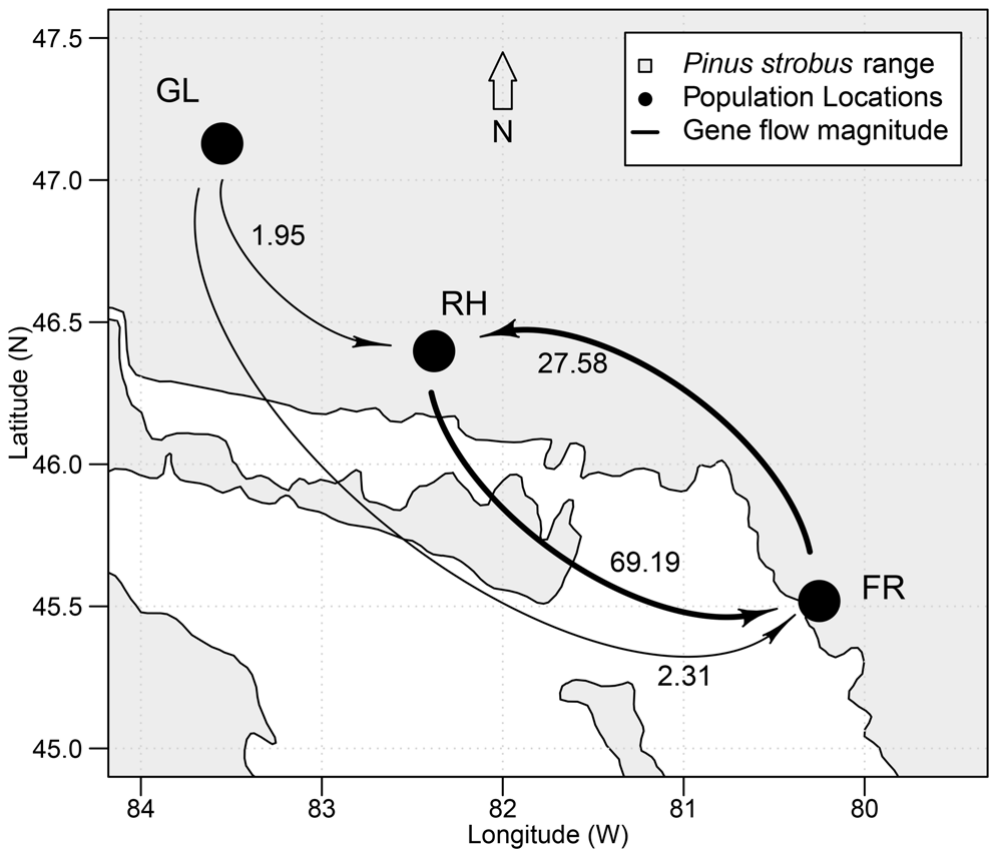
Gene flow between three study sites under north to south (marginal to central) gene flow model. The north to south gene flow model assumed that the central populations exchange migrants freely. The numbers on arrows represent number of immigrants per generation.

Of the three gene flow models tested, the north to south (marginal to central) model had the highest probability (*P* = 1.00) based on the comparison of marginal likelihood Bayes factors (Table S4). The number of migrants per generation received from northern populations is depicted in Fig. 5. Although this model allowed for free exchange of migrants between the two central locations, French River population still received substantially more migrants from Rawhide Lake population than *vice-versa*. Estimates of migration parameters for individual populations were well within the confidence intervals for the tested model. The observed pattern of gene flow is probably a result of the prevailing north to south wind flow in the study area.

## Discussion

### Genetic Diversity and *N*_e_

Our results demonstrate that EWP central populations have significantly higher allelic diversity, genotypic diversity (*GA*_e_), *N*_e_, and LGP than the marginal populations but statistically similar heterozygosity and *F*_IS_. These patterns were consistent when two OG marginal populations were compared with two OG central or two SG central populations (Table 2). The same pattern for allelic diversity and *H*_e_ was also observed for the same GL and FR populations from allozyme analysis (Rajora, unpublished data).

The lower allelic diversity in marginal than central populations may be a result of one or more factors and their interactions, such as small census size and *N*_e_, isolation, bottleneck, and higher genetic drift and founder effect in marginal than central populations. Both marginal GL populations are isolated, whereas the central RH and FR populations are continuous. The *N*_e_ estimates for the marginal populations are significantly lower than those of the central populations (Table 2).

Geological, pollen and macrofossil data suggest an abundance of EWP in southern Ontario and southern Quebec above its current range limit during Holocene period between 9,000 and 5,000 years before present (BP) [24], [61], indicating the presence of warmer climate more favorable for range expansion than at present. Cooler climate set in somewhere between 3,000 to 2,000 years BP [24]. Marginal GL populations possibly experienced bottleneck, founder effect and fluctuations of *N*_e_ resulting from this post-glaciation range expansion and retraction during the onset of colder climate. These factors will enhance genetic drift in the marginal populations, which will reduce genetic diversity, especially allelic diversity. Divergent selection in conjunction with drift can also reduce allelic diversity [62]. We observed signatures of divergent selection at two loci in the marginal populations. Model testing suggested that gene flow to the marginal populations was likely curtailed.

The similar levels of heterozygosity in central and marginal populations may be due to longevity of EWP, loss of very little heterozygosity due to bottleneck and genetic drift over a few generations, and existence of moderate number of individuals in marginal populations. Genetic bottlenecks can disrupt the mutation-drift equilibrium in natural populations [62], which reduces genetic diversity due to genetic drift, depleting allelic diversity much faster than heterozygosity [27], [31], [63], [64]. Eastern white pine can live more than 400 years, and the sampled marginal populations had moderate to large number of individuals. The maintenance of heterozygosity in marginal populations may also be due to more pronounced heterozygote advantage in sub-optimal environmental conditions [64]. The marginal GL populations experience harsh climate and site conditions in terms of temperature and ecosite characteristics as discussed below.

The patterns of genetic diversity (allelic diversity and heterozygosity) and *N*_e_ between central and marginal populations are consistent with those recently reported for long-lived conifer, *Thuja occidentalis* [11], [12]. Our results support the central-marginal hypothesis. However, the results are in contrast with the genetic diversity results previously reported for central Ontario and marginal Newfoundland populations of EWP [13]. This inconsistency may be due to differences in the parts of the range sampled, markers used and sampling design. Rajora *et al*. [13] study was based on selective sampling, allozyme markers and populations separated by about 2,000 km, whereas our study was based on population census, microsatellite markers and sampling from the same part of EWP range in northern Ontario. Also, our results are in contrast to some studies in other conifers that reported similar genetic diversity between marginal and central populations, e.g., [15].

The studied old-growth and second-growth EWP populations have similar levels of genetic diversity, *N*_e_, inbreeding and LGP. These results are in contrast with the general belief and limited empirical evidence [20], [21] that old-growth populations have higher genetic diversity than the second-growth populations, but are in agreement with [28], who reported similar genetic diversity for one old-growth and one second-growth EWP stand from USA. Old-growth stands are expected to harbor higher genetic diversity due to genetic homeostasis, selection against inbreds and survival of the fittest over time in long-lived plants. The age of the studied old-growth populations was ∼250 years and that of second-growth populations ∼100 years, similar to that sampled in [28]. We do not know the exact reasons for similar genetic diversity and *N*_e_ observed in OG and SG populations. It may be possible that EWP goes through the processes that maintain high genetic diversity by age 100 years. EWP has predominantly outcrossing mating system [14], [25] and severe inbreeding depression where, like other conifers [65], selection against inbreds probably occurs at a very early stage. These features will help in genetic diversity maintenance in ∼100 years old SG EWP.

We sampled central and marginal and old-growth and second-growth populations from a relatively small part of EWP range in northern Ontario in order to avoid major regional variation confounding our results. Study of populations from other parts of EWP range may yield different results. Also, a range-wide central-marginal population genetic study in EWP is needed.

### Population Structure and Genetic Divergence

Bayesian structure, *F*_ST_, AMOVA, genetic distance, NJ, and PCA consistently demonstrated that the marginal populations are genetically diverged and separated from the central populations. This may be a result of isolation, impeded gene flow, genetic drift and divergent selection and their synergistic effects. Although different Mantel tests were not significant, the isolation by distance (IBD) contributing to the genetic differentiation of the marginal populations from the central populations could not be completely ruled out due to low statistical power to detect IBD with a limited number of populations examined in the present study. Nevertheless, other genetic differentiation measures such as AMOVA attributed hierarchical population structure to the divergence of marginal populations where a change in allele frequencies towards fixation or purging was observed at loci RPS-1b, RPS-20, RPS-34b and RPS-39. Also curtailed gene flow to marginal populations (Fig. 5) and divergent selection at RPS-20 and RPS-39 (Fig. 3) were observed. Thus, genetic drift coupled with limited gene flow from central populations and possibly divergent selection seems to be the most plausible explanation for the genetic distinctness of the marginal populations. We also observed that EWP central old-growth populations are genetically similar to central second-growth populations. This may be due to large population size, continuous distribution and long-distance gene flow.

Marginal populations are expected to have higher differentiation among themselves than central populations [1], [2]. While there is empirical support for this view [1], [2], [11], we did not find strong support for this pattern. We attribute these results to the geographical proximity of the two marginal GL populations, which are separated by about 2 km. Although these populations have private alleles [27], [31], there are no known geographical barriers to gene flow between them. Likewise, our study suggests very little genetic differentiation between 190 km apart central OG and SG populations. This is consistent with the expectation for central populations of highly outcrossing species with long-distance gene flow.

### Curtailed Asymmetrical Gene Flow to Marginal Populations

The Bayesian model-based approach demonstrated that north to south (marginal to central) migration was the most likely model explaining the observed data. This pattern coincides with the prevalent north to south wind flow in the area. Geographical isolation of GL populations also may have resulted in restricted gene flow to them. Thus the observed pattern of migration is in agreement with the theoretical central-marginal expectation of curtailed gene flow to marginal populations despite abundant center. Coalescent methods, such as MIGRATE, estimate migration rates accurately when the population divergence time is deep. Because the time of population split between the studied central and marginal populations is likely to be very recent (<200 generations) based on the geological and fossil evidence, some assumptions have likely been violated in the gene flow analysis using MIGRATE. Thus caution must be exercised when interpreting the rates of migration between the study populations.

### Signatures of Divergent and Balancing Natural Selection in Marginal Populations

Our study provides molecular evidence for divergent and balancing selection operating in the marginal populations of EWP in northern Ontario. Two alleles at RPS-20 and four alleles at RPS-39, candidate loci for divergent selection, showed sharp directional change in their frequencies in the marginal populations (Fig. 4). Such a dramatic difference in gene frequencies may also result from strong genetic drift in marginal populations where rare alleles from core populations may surf the wave of population expansion and greatly increase in frequency. This phenomenon, dubbed as ‘allele surfing’ [66] has been found to account for many of the large gene frequency differences in human populations from different continents, previously attributed to local adaptation through selection following migration out of Africa [67]. We do not find allele surfing contributing to allele frequency spikes at RPS-20 and RPS-39 on account of several reasons. First, our marginal populations had once been a part of the continuous distribution of the species, which went through multiple cycles of range expansion and contraction, a proposition supported by fossil pollen evidence [24]. Therefore, the Galloway Lake marginal populations do not represent the *de-novo* leading edge of eastern white pine range expansion, a characteristic target of allele surfing [66]. Second, the probability of a rare allele to surf increases with the reduction in the size and connectivity (through gene flow) between the local demes [67] (and references therein), for which we found no evidence between the two marginal populations. Finally, surfing is expected to cause multiple rare alleles to increase in frequency in an expanding population [67]. In our study, increase in the frequency of rare alleles in the marginal populations was not across the loci but was limited to only one allele at RPS-20 and two alleles at RPS-39 (data for other loci not shown).

The divergent selection in the marginal populations is likely due to their local adaptation to different climatic and site conditions. The studied marginal and central EWP populations occur in two different ecoregions of Ontario. The GL populations occur in the ecoregion 4E (Tamagami Ecoregion) and the central RH and FR populations in the ecoregion 5E (Georgian Bay Ecoregion) [33]. The ecoregion 4E is characterized as Humid Low Boreal Ecoclimatic Region, whereas the ecoregion 5E as Humid High Moderate Ecoclimatic Region [33]. These ecoregions differ in mean annual temperatures, average growing season, rainfall and climate [33], with ecoregion 4E (and marginal populations therein), experiencing harsher climate and site conditions. Such climatic and ecological differences could result in different selection regimes in the GL populations thereby driving changes in frequencies of alleles. The selection pressures and regimes are most likely to be different in different parts of the geographical distribution range of EWP which has wide geographical distribution in North America encountering a variety of temperature, moisture, soil and other ecological conditions in its central and marginal populations. Under this scenario, the loci showing signatures of selection in marginal populations from different parts of the EWP range are likely to be different. The studied populations are from the northern Ontario part of the distribution range of EWP. Thus, broad inferences about range-wide selection pressures and loci showing signatures of selection in marginal populations cannot be drawn here. Nevertheless, our results provide robust inference of genetic divergence and natural selection in EWP marginal populations in the part of the EWP range in northern Ontario that we studied, where marginal populations are expected to expand northward.

The GL marginal populations demonstrated the presence of divergent selection at two candidate loci under both simple and hierarchical island models (Fig. 3; Fig. S4). Therefore, it is unlikely that the effects of shared population history or presence of hierarchical population structure have confounded the effects of natural selection on these loci. Moreover, our results of divergent selection in marginal populations are consistent with similar results reported for SNP markers in *Picea sitchensis* [18] and for AFLP markers in *Buscutella laevigata* [19].

Under balancing selection, heterozygotes of beneficial alleles may be maintained preferentially over homozygotes. Indeed, for RPS-12 consistently showing the signatures of balancing selection, *H*_o_ was high in marginal populations. The balancing selection may be due to heterozygote advantage in marginal harsher climatic conditions [64] that the sampled marginal populations experience.

Signatures of selection in response to salt tolerance have been reported for microsatellite markers in *Helianthus paradoxus* [68]. Microsatellite genetic variation is generally assumed to be selectively neutral. However, it is possible that the selective candidate microsatellite markers are in linkage disequilibrium with functional causative variation in the genome, responsible for local adaptation in marginal populations. This proposition is supported by the fact that none of the three candidate loci were detected to be under selection when only central OG and SG populations were analyzed. The status of these markers will remain putative until linkage of these microsatellite loci with adaptive variation (e.g. SNPs) is ascertained. Nevertheless, we believe that our study provides the first evidence for divergent and balancing natural selection operating in *in-situ* natural marginal populations of long-lived and widely distributed plants.

Although SNPs in candidate genes and genomic elements/sequences make powerful markers to detect natural selection, microsatellites provide more precise information on genetic diversity, population structure and demography [69], which is essential for disentangling the confounding effects of demographic processes and shared population history from that of selection. Microsatellite markers have allowed us to address the primary objective of examining differences in genetic diversity and population structure between central and marginal EWP populations from northern Ontario. In future, a large number of SNPs in candidate genes and other genomic elements along with microsatellites should be used to identify genes under selection in marginal populations.

## Conclusions

Our study results conform to most of the theoretical expectations of central-marginal hypothesis. The marginal EWP populations have lower allelic diversity, *N*_e_ and LGP than the central populations. The marginal populations have slightly higher but statistically similar heterozygosity to the central populations. The central and marginal EWP populations have similar levels of genetic divergence. The marginal EWP populations are genetically distinct from the central populations. Gene flow is asymmetrical with north to south migration fitting the observed data better than either south to north or panmixia models, consistent with the prevailing north-south wind pattern in the area. The marginal populations showed signatures of diversifying and balancing selection, probably in response to local adaptation. Curtailed gene flow and natural selection may be potential mechanisms underlying local adaptation of the GL marginal populations. The successional stage (old-growth and second-growth) of the populations apparently has no effect on the central-marginal population genetic patterns in EWP. The studied EWP old-growth and second-growth populations have similar genetic diversity and genetic constitution. Our study provides the original report on the dynamics of migration, genetic drift and selection in central and marginal populations of EWP, and perhaps of any long-lived plant species with wide geographical distribution. The results contribute to resolving the classical central-marginal debate and to the understanding of evolutionary genetic forces underlying local adaptation in marginal populations.

Conservation value of marginal populations is debatable. We strongly caution against discounting the genetic conservation importance of the Galloway Lake area EWP populations, considering their genetic distinctness and potential for evolutionary change and local adaptation for future range expansion under anticipated climate change conditions. Our study calls for more extensive range-wide investigations of genome-wide and adaptive genetic variation and population structure in EWP, which could offer insights into local adaptation of the marginal populations and effectively address conservation genetic issues.

## Supporting Information

Figure S1 **Neighbor-Joining tree showing genetic relationships among central and marginal populations of eastern white pine.**
(PDF)

Figure S2 **Principal coordinates plot showing genetic relationships of eastern white pine populations.** Ordination of the eastern white pine populations on principal coordinates 1 (PC1) and 2 (PC2) based on their Nei (1972) genetic distances.
(PDF)

Figure S3 **Log probability of data and Δ** ***K*** **estimates for all central and marginal eastern white pine populations.**
(PDF)

Figure S4 ***F*** **_ST_ outliers showing signatures of natural selection under simple island model.** *F*_ST_ outlier graphs showing microsatellite loci putatively under selection in **(a)** central *vs* marginal populations and, **(b**) central old-growth *vs* central second-growth populations.
(PDF)

Figure S5 ***F*** **_ST_ outlier test for old-growth populations under simple island model.** *F*_ST_ outlier test for detection of candidate loci under selection in two old-growth marginal and two old-growth central populations.
(PDF)

Figure S6 ***F*** **_ST_ outlier test for three pooled populations under simple island model.** *F*_ST_ outlier test for detection of candidate loci under selection in three pooled central and marginal populations of eastern white pine.
(PDF)

Figure S7 **Detection of natural selection using Bayesian** ***F*** **_ST_ method. Bayesian analysis for detection of candidate loci under selection in central and marginal populations.** ***F*** **_ST_ is plotted against log of posterior probability** ***q*** **values that indicate outlier status of markers. Filled circles represent markers under selection.**
(PDF)

Figure S8 **Effective population sizes and their 95% confidence intervals for eastern white pine populations.** Effective population size estimates for six individual populations (A), and for three pooled populations (B). The K numbers indicate MCMC sweeps discarded as burn-in and recorded respectively.
(PDF)

Table S1 (DOCX)

Table S2 (DOCX)

Table S3 (DOCX)

Table S4 (DOCX)

Microsatellite Genotyping Text S1 (DOCX)
