# Supplementary figures and images for: Genetic Divergence and Signatures of Natural Selection in Marginal Populations of a Keystone, Long-Lived Conifer, Eastern White Pine (Pinus strobus) from Northern Ontario
Source: PLoS One. 2014 May 23;9(5):e97291. doi: 10.1371/journal.pone.0097291 (PMC4032246; doi:10.1371/journal.pone.0097291)

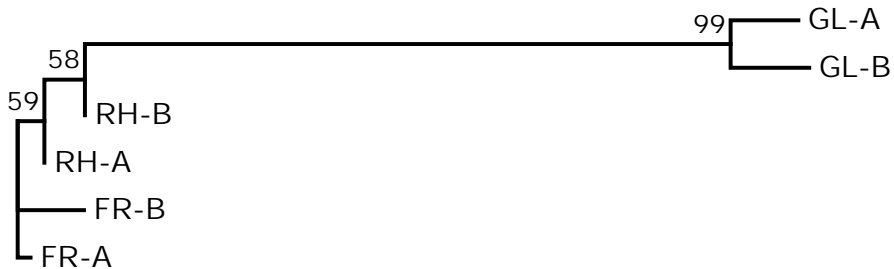

0.02

Supplement: Figure S1 — Neighbor-Joining tree showing genetic relationships among central and marginal populations of eastern white pine. (PDF) [file pone.0097291.s001.pdf]

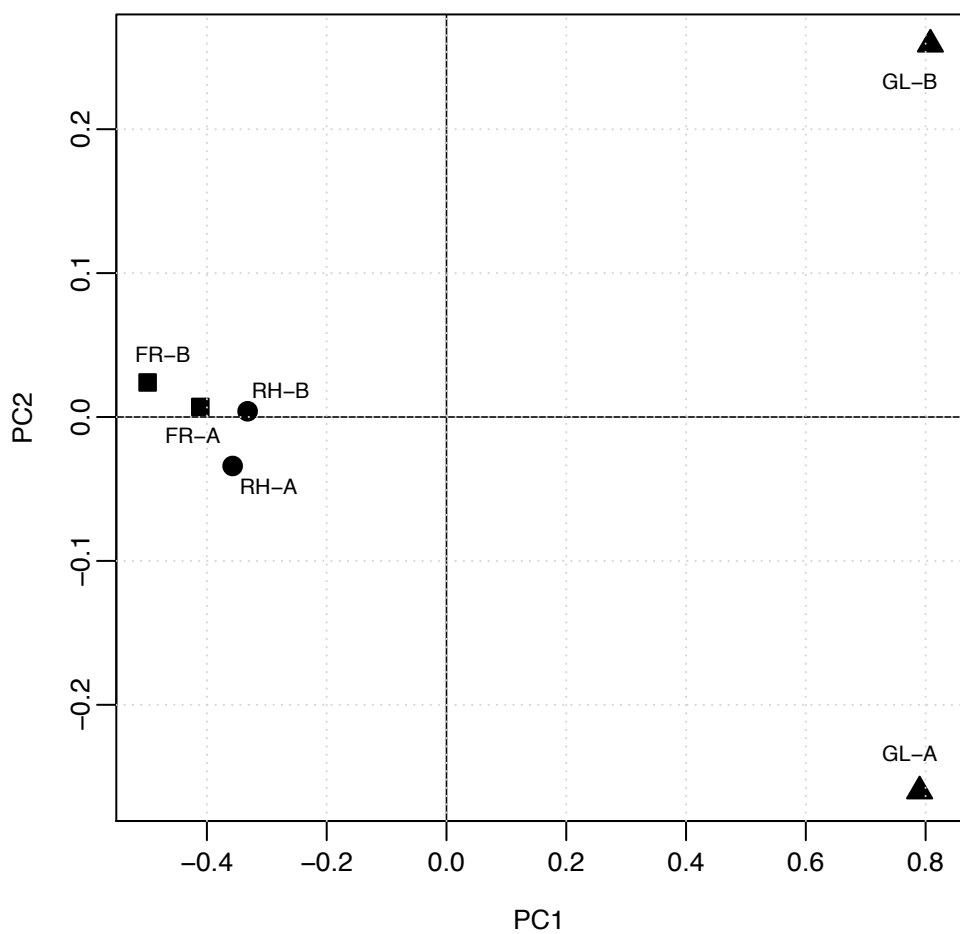

Supplement: Figure S2 — Principal coordinates plot showing genetic relationships of eastern white pine populations. Ordination of the eastern white pine populations on principal coordinates 1 (PC1) and 2 (PC2) based on their Nei (1972) genetic distances. (PDF) [file pone.0097291.s002.pdf]

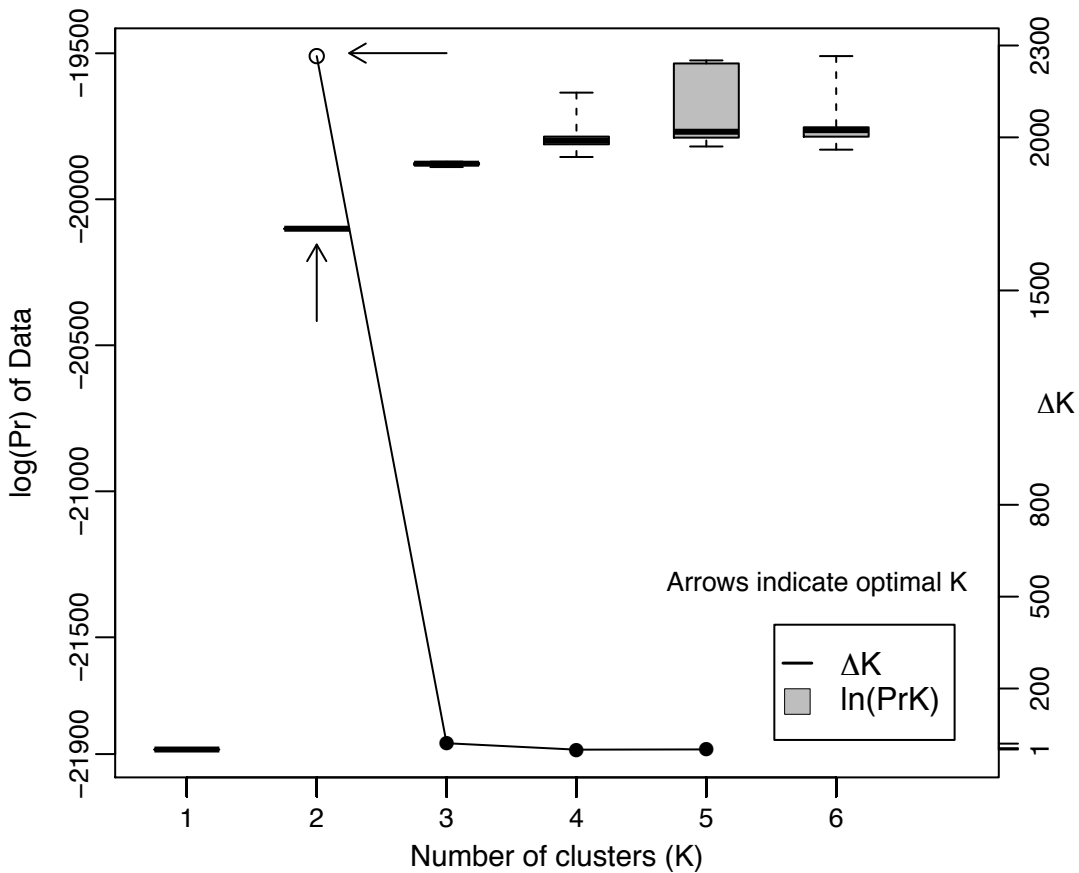

Supplement: Figure S3 — Log probability of data and Δ K estimates for all central and marginal eastern white pine populations. (PDF) [file pone.0097291.s003.pdf]

(a)

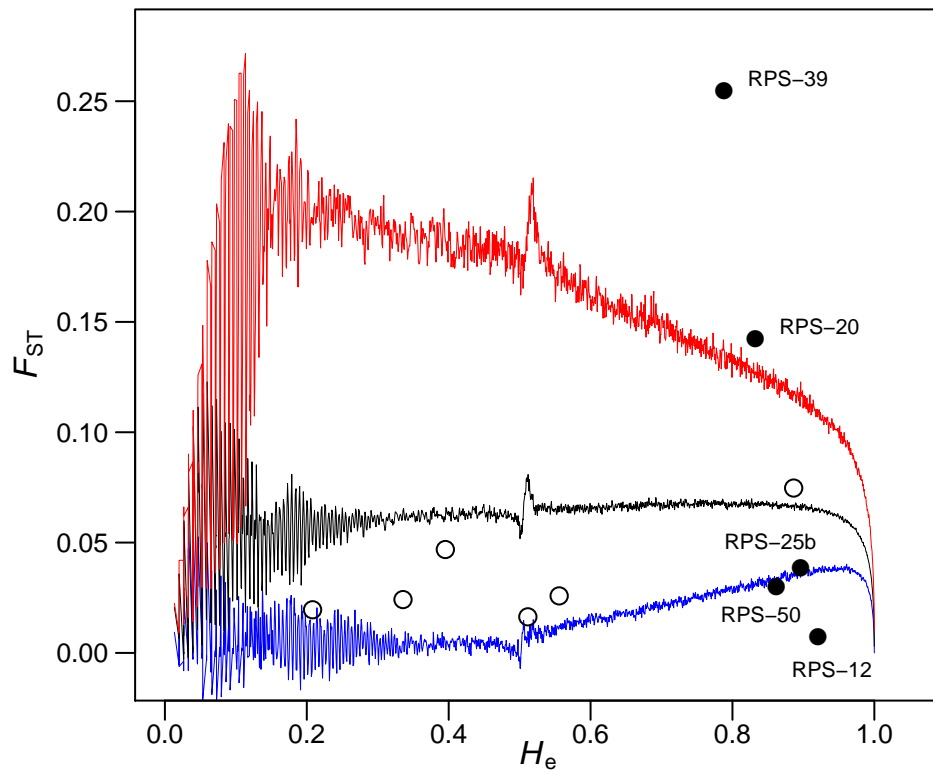

(b)

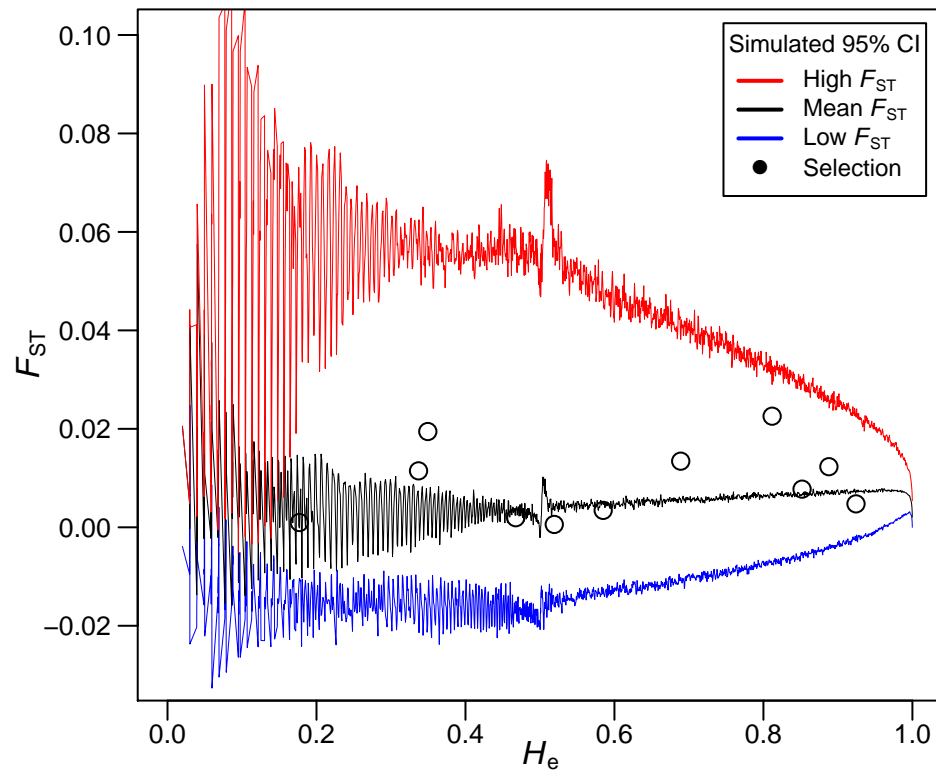

Supplement: Figure S4 — F ST outliers showing signatures of natural selection under simple island model. F ST outlier graphs showing microsatellite loci putatively under selection in (a) central vs marginal populations and, (b) central old-growth vs central second-growth populations. (PDF) [file pone.0097291.s004.pdf]

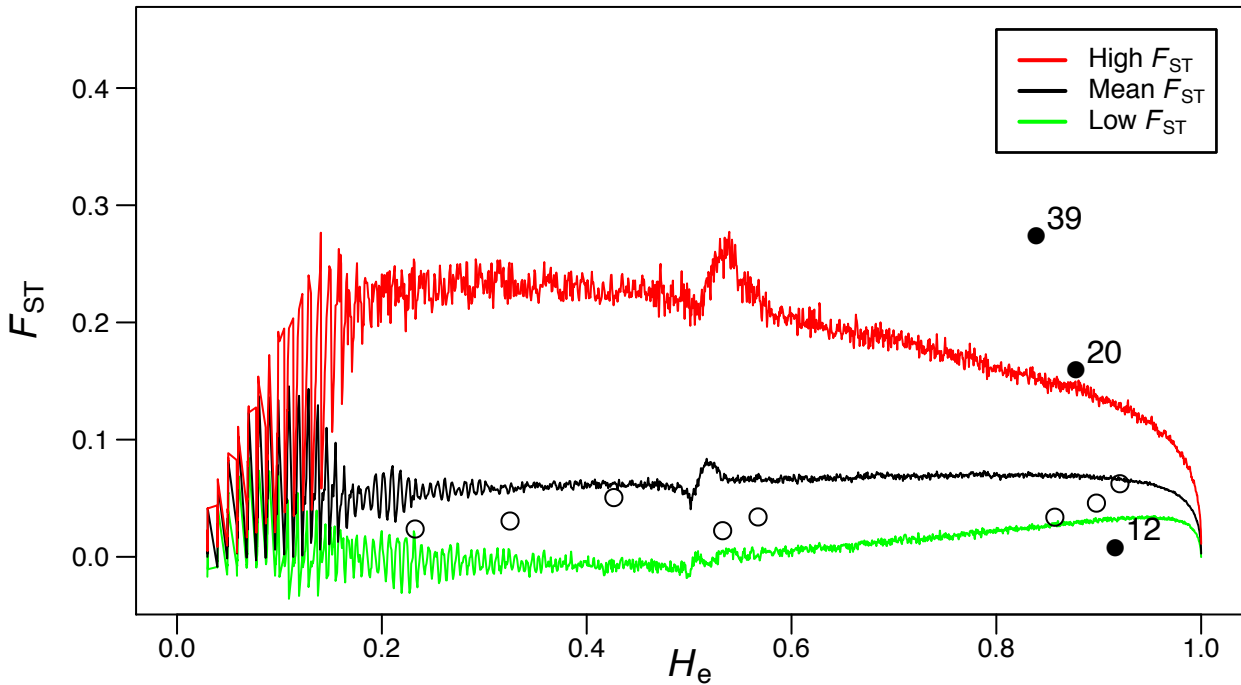

Supplement: Figure S5 — F ST outlier test for old-growth populations under simple island model. F ST outlier test for detection of candidate loci under selection in two old-growth marginal and two old-growth central populations. (PDF) [file pone.0097291.s005.pdf]

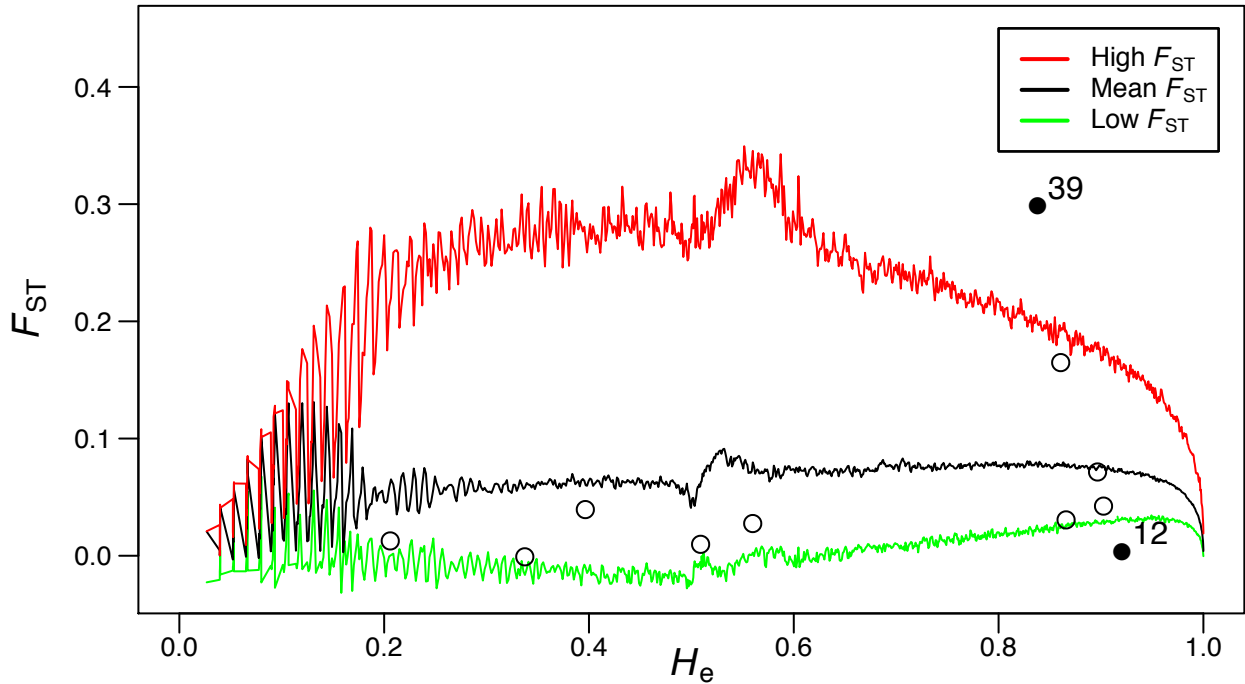

Supplement: Figure S6 — F ST outlier test for three pooled populations under simple island model. F ST outlier test for detection of candidate loci under selection in three pooled central and marginal populations of eastern white pine. (PDF) [file pone.0097291.s006.pdf]

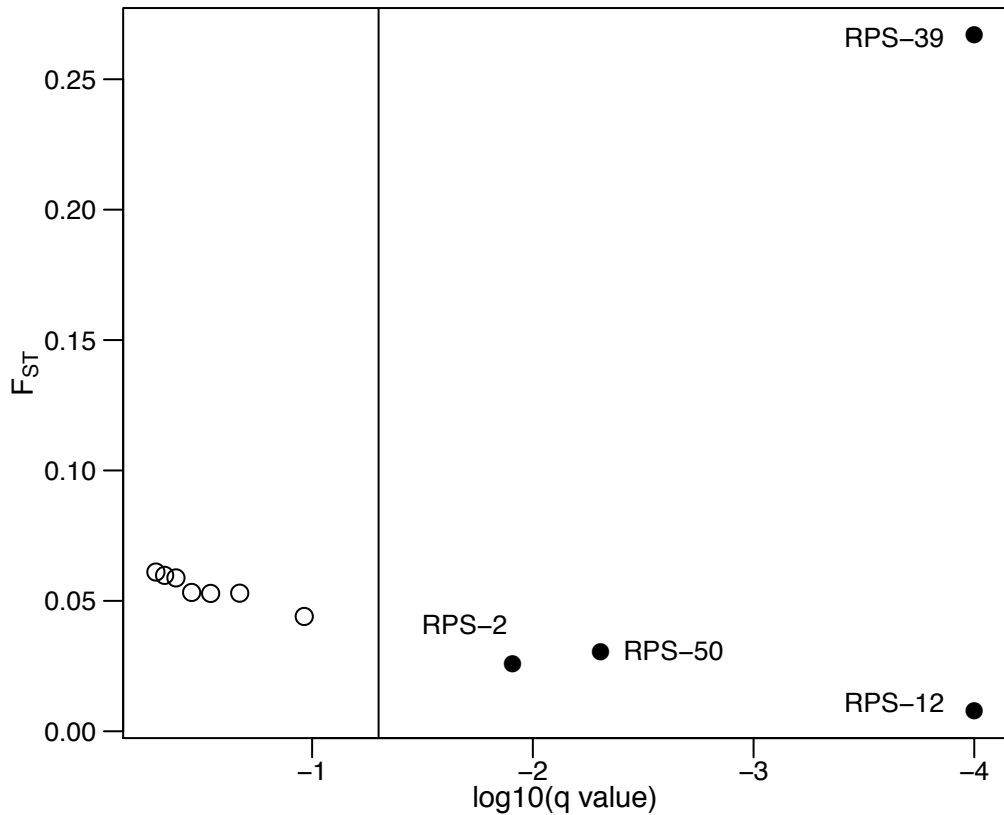

Supplement: Figure S7 — Detection of natural selection using Bayesian F ST method. Bayesian analysis for detection of candidate loci under selection in central and marginal populations. F ST is plotted against log of posterior probability q values that indicate outlier status of markers. Filled circles represent markers under selection. (PDF) [file pone.0097291.s007.pdf]

# $N_e$ Estimates and 95% Confidence Intervals

**(a) Individual Populations**

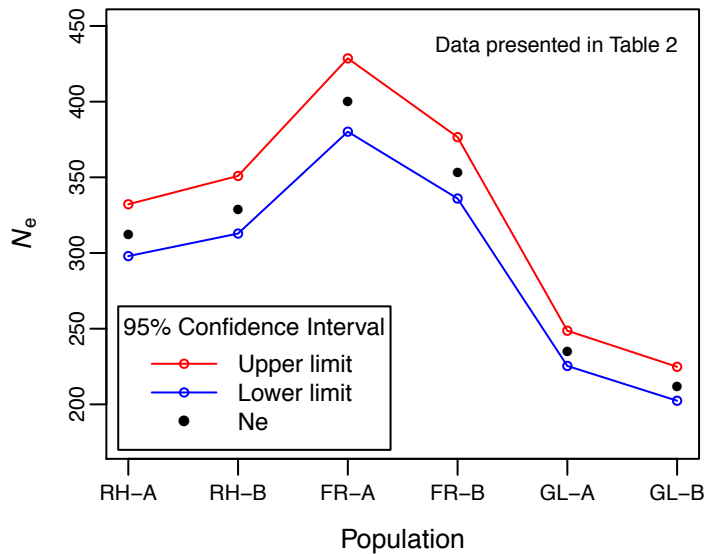

**(b) Pooled Populations**

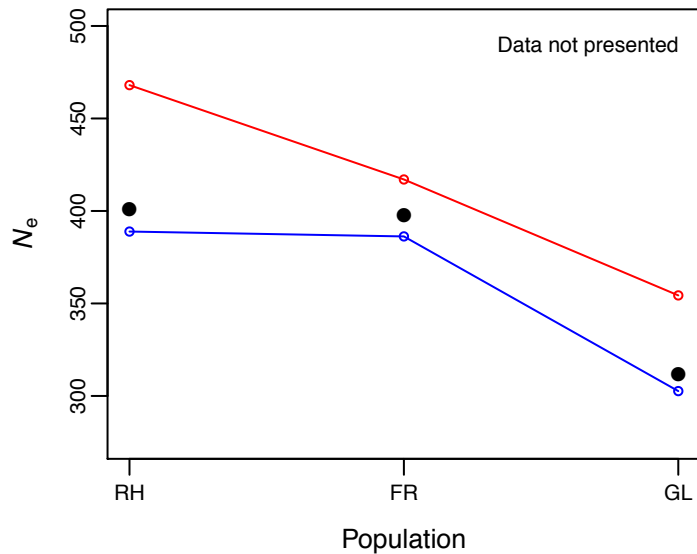

Supplement: Figure S8 — Effective population sizes and their 95% confidence intervals for eastern white pine populations. Effective population size estimates for six individual populations (A), and for three pooled populations (B). The K numbers indicate MCMC sweeps discarded as burn-in and recorded respectively. (PDF) [file pone.0097291.s008.pdf]
