# Supplementary material for: Genetic Divergence and Signatures of Natural Selection in Marginal Populations of a Keystone, Long-Lived Conifer, Eastern White Pine (Pinus strobus) from Northern Ontario
Source: PLoS One. 2014 May 23;9(5):e97291. doi: 10.1371/journal.pone.0097291 (PMC4032246; doi:10.1371/journal.pone.0097291)
Supplement: Table S1 — (DOCX) [file pone.0097291.s009.docx]

**Table S1** Number of alleles and their size range at individual microsatellite loci

| Locus | Repeat Type | Number of Alleles | Size Range (bp) |
| --- | --- | --- | --- |
| RPS-1b | (AC)10 | 13 | 191-217 |
| RPS-2 | (AC)10 | 13 | 145-173 |
| RPS-12 | (AC)17 | 36 | 149-229 |
| RPS-20 | (AC)16(AT)6 | 26 | 100-180 |
| RPS-25b | (AC)15 | 28 | 81-163 |
| RPS-34b | (AC)14 | 12 | 141-171 |
| RPS-39 | (AC)17 | 11 | 158-188 |
| RPS-50 | (AC)17 | 18 | 152-192 |
| RPS-118b | (AC)23 | 23 | 124-168 |
| RPS-119 | (AC)10(AT)10 | 2 | 203-205 |
| RPS-127 | (AC)10(AT)5 | 3 | 191-195 |
