# Supplementary material for: Genetic Divergence and Signatures of Natural Selection in Marginal Populations of a Keystone, Long-Lived Conifer, Eastern White Pine (Pinus strobus) from Northern Ontario
Source: PLoS One. 2014 May 23;9(5):e97291. doi: 10.1371/journal.pone.0097291 (PMC4032246; doi:10.1371/journal.pone.0097291)
Supplement: Table S2 — (DOCX) [file pone.0097291.s010.docx]

**Table S2** Pair-wise *F*_ST_ (lower diagonal) and genetic distances (Nei 1972) (upper diagonal) between eastern white pine populations

| Population | RH-A | RH-B | FR-A | FR-B | GL-A | GL-B |
| --- | --- | --- | --- | --- | --- | --- |
| RH-A |  | 0.010 | 0.006 | 0.013 | 0.206 | 0.222 |
| RH-B | 0.006 |  | 0.021 | 0.016 | 0.209 | 0.222 |
| FR-A | 0.003 | 0.012 |  | 0.015 | 0.236 | 0.248 |
| FR-B | 0.010 | 0.011 | 0.011 |  | 0.249 | 0.260 |
| GL-A | 0.104 | 0.104 | 0.114 | 0.133 |  | 0.039 |
| GL-B | 0.107 | 0.106 | 0.115 | 0.134 | 0.021 |  |
