# Supplementary material for: Genetic Divergence and Signatures of Natural Selection in Marginal Populations of a Keystone, Long-Lived Conifer, Eastern White Pine (Pinus strobus) from Northern Ontario
Source: PLoS One. 2014 May 23;9(5):e97291. doi: 10.1371/journal.pone.0097291 (PMC4032246; doi:10.1371/journal.pone.0097291)
Supplement: Table S3 — (DOCX) [file pone.0097291.s011.docx]

**Table S3** Functional annotation of eastern white pine microsatellites-containing genomic sequences with protein BlastX

| Locus | Selection | GID | Top Hits | Organism | Protein | Cov | EV | MID |
| --- | --- | --- | --- | --- | --- | --- | --- | --- |
| RPS-1b |  | U60239 | XP-001310379.1 | *Trichomonas vaginalis* | TVAG-226930 | 25 | 2.9 | 55 |
| RPS-2 |  | U60240 | No hits |  |  |  |  |  |
| RPS-12 | Balancing | U60242 | ZP-02163267.1 | *Kordia algicida* | KAOT1-09806 | 20 | 2.9 | 64 |
|  |  |  | EFN85188.1 | *Harpegnathos saltator* | EAI-06382 | 44 | 8.4 | 32 |
| RPS-20 | Divergent | U60244 | CBH16565.1 | *Trypanosoma brucei* | Hypo. protein | 92 | 6.6 | 33 |
| RPS-25b |  | U60245 | No hits |  |  |  |  |  |
| RPS-34b |  | U60246 | No hits |  |  |  |  |  |
| RPS-39 | Divergent | U60247 | No hits |  |  |  |  |  |
| RPS-50 |  | U60248 | No hits |  |  |  |  |  |
| RPS-118b |  | U60252 | No hits |  |  |  |  |  |
| RPS-119 |  | U60253 | EGD80719.1 | *Salpingoeca sp.* | PTSG-11705 | 60 | 4.1 | 72 |
|  |  |  | XP-003318567.1 | *Pan troglodytes* | LOC100611980 | 39 | 3 | 72 |
|  |  |  | XP-671115.1 | *Plasmodium berghei* | Hypo. protein | 37 | 1.5 | 58 |
|  |  |  | XP-003084714.1 | *Mus musculus* | Hypo. protein | 34 | 2.4 | 81 |
| RPS-127 |  | U60255 | EGD80719.1 | *Salpingoeca sp.* | PTSG-11705 | 60 | 4.1 | 72 |
|  |  |  | XP-003318567.1 | *Pan troglodytes* | LOC100611980 | 39 | 3 | 72 |
|  |  |  | XP-671115.1 | *Plasmodium berghei* | Hypo. protein | 37 | 1.5 | 58 |

GID: Genbank ID, Cov: Percent coverage (%), EV: E value, MID: Maximum identity (%)
