# Supplementary material for: Genetic Divergence and Signatures of Natural Selection in Marginal Populations of a Keystone, Long-Lived Conifer, Eastern White Pine (Pinus strobus) from Northern Ontario
Source: PLoS One. 2014 May 23;9(5):e97291. doi: 10.1371/journal.pone.0097291 (PMC4032246; doi:10.1371/journal.pone.0097291)
Supplement: Table S4 — (DOCX) [file pone.0097291.s012.docx]

**Table S4** Marginal likelihood and probability for various gene flow models

| Model | Log(ML)^1^ | LBF^2^ | Model-Probability |
| --- | --- | --- | --- |
| Full | -104892.12 | -82336.53 | 0.0000 |
| South to North | -98155.91 | -75600.32 | 0.0000 |
| North to South | -22555.59 | 0.00 | 1.00 |

^1^Log (Marginal Likelihood)

^2^Log10 Bayes Factors
