# Supplementary material for: Genetic Divergence and Signatures of Natural Selection in Marginal Populations of a Keystone, Long-Lived Conifer, Eastern White Pine (Pinus strobus) from Northern Ontario
Source: PLoS One. 2014 May 23;9(5):e97291. doi: 10.1371/journal.pone.0097291 (PMC4032246; doi:10.1371/journal.pone.0097291)
Supplement: Microsatellite Genotyping Text S1 — (DOCX) [file pone.0097291.s013.docx]

**Supporting Information**

**Genetic divergence and signatures of natural selection in marginal populations of a keystone, long-lived conifer, eastern white pine (*Pinus strobus*) from northern Ontario**

VIKRAM E. CHHATRE1,3 and OM P. RAJORA1,2*

1Department of Biology, Dalhousie University, Halifax, Nova Scotia B3H 4J1, Canada

2Faculty of Forestry and Environmental Management, University of New Brunswick, 28 Dineen Drive, Fredericton, New Brunswick E3B 5A3, Canada

3Present Address: Southern Institute of Forest Genetics, USDA Forest Service, Southern Research Station, 23332 Success Road, Saucier, MS 39574, USA

*Author for correspondence

Om P. Rajora

Faculty of Forestry and Environmental Management

University of New Brunswick, 28 Dineen Drive,

Fredericton, New Brunswick E3B 5A3, Canada

E-mail: [Om.Rajora@unb.ca](mailto:Om.Rajora@unb.ca)

Fax : (506) 453-3538

**Supplementary Text S1**

**Microsatellite Genotyping**

PCR amplification was carried out in a total volume of 10μL containing about 40 ng of template DNA, 1.5–2.5 mM MgCl2, 2.5 mM dNTP mix, 250 nM of each of the forward and reverse primers, 0.5 U of Taq DNA polymerase (Invitrogen Corp., Burlington, ON, Canada) and 10X PCR buffer (750 mM Tris-HCl, 200 mM (NH4)2SO4, 0.1% Tween 20). The 5’ labeled forward primers with IRDYE-700 or IRDYE-800 were obtained from MWG Biotech (High Point, NC, USA). All PCR reactions were performed in PTC-200 thermal cyclers (MJ Research, Bruno, QC, Canada) using the following profile: an initial denaturation step of 3 min at 94◦C, 2 cycles with a denaturation for 15 sec at 94◦C, annealing for 15 sec at 60◦C and extension for 15 sec at 72◦C. This was followed by a touchdown protocol that reduced the annealing temperature from 60◦C to 54◦C in 11 cycles comprising of extension for 15 sec at 72◦C. This was further followed by 25–29 cycles comprising denaturation for 15 sec at 94◦C, annealing for 15 sec at 54◦C and an extension for 15 sec at 72◦C. There was a 3-min final extension step at 72◦C.

One μL PCR product was diluted in 15–30 μL of loading buffer (47.5 mL of deionized Formamide, 4 mL of 0.5M EDTA and 25mg Bromophenol blue in a total of 50 mL of loading buffer). The PCR products were denatured for 3 minutes at 94◦C before loading on 6% denaturing Polyacrylamide gel (7M Urea). The electrophoresis was performed using Global IR2 4200 DNA Sequencer (LI-COR Biosciences, Lincoln, Nebraska) for 1 hour 30 minutes at a constant voltage of 1500V. The electrophoresed PCR products were analyzed with a 50–350 bp DNA molecular weight standard (LI-COR). Genotypes of all samples were first scored using LI-COR’s SAGA/GT- MX software suite for microsatellite analysis and then verified manually.
